# The Reproducibility of the Immunohistochemical PD-L1 Testing in Non-Small-Cell Lung Cancer: A Multicentric Italian Experience

**DOI:** 10.1155/2019/6832909

**Published:** 2019-04-14

**Authors:** Elena Vigliar, Umberto Malapelle, Francesca Bono, Nicola Fusco, Diego Cortinovis, Emanuele Valtorta, Alexiadis Spyridon, Manuela Bimbatti, Mario Zocchi, Chiara Piva, Gabriella Gaudioso, Antonino Iaccarino, Patrizia Morbini, Fabio Pagni

**Affiliations:** ^1^Department of Public Health, University Federico II of Naples, Naples, Italy; ^2^San Gerardo and Desio Hospital, ASST Monza, Pathology, Monza, Italy; ^3^Division of Pathology, Fondazione IRCCS Ca' Granda, Ospedale Maggiore Policlinico, University of Milan, Milan, Italy; ^4^San Gerardo Hospital, Oncology, Monza, Italy; ^5^Niguarda Cancer Center, Grande Ospedale Metropolitano Niguarda, Milano, Italy; ^6^Human Pathology and Molecular Pathology Unit, San Paolo Hospital, Department of Health Sciences, Università degli Studi di Milano, Milan, Italy; ^7^Division of Pathology, Department of Health Sciences, University of Eastern Piedmont, Novara, Italy; ^8^Pathology Unit, Policlinico S. Matteo, Pavia, Italy; ^9^University Milan Bicocca, Department of Medicine and Surgery, Milan, Italy

## Abstract

An important harmonization effort was produced by the scientific community to standardize both the preanalytical and interpretative phases of programmed death-ligand 1 (PD-L1) immunohistochemical (IHC) testing in non-small-cell lung cancer (NSCLC). This analysis is crucial for the selection of patients with advanced-stage tumors eligible for treatment with pembrolizumab and potentially with other anti-PD-1/PD-L1 checkpoint inhibitors. This multicentric retrospective study evaluated the reproducibility of PD-L1 testing in the Italian scenario both for* closed* and* open* platforms. In the evaluation of the well-known* gold-standard* combinations (Agilent 22C3 PharmDx on Dako Autostainer versus Roche's Ventana SP263 on BenchMark), the results confirmed the literature data and showed complete overlapping between the two methods. With regard to the performances by using* open* platforms, the combination of 22C3 with Dako Omnis or Benchmark obtained good results basically, while the 28,8 clone seemed to be associated with worse scores.

## 1. Introduction

An important harmonization effort was produced by the scientific community to standardize both the preanalytical and interpretative phases of programmed death-ligand 1 (PD-L1) immunohistochemical (IHC) testing in non-small-cell lung cancer (NSCLC) [1, 2]. This analysis is crucial for the selection of patients with advanced-stage tumors eligible for treatment with pembrolizumab and potentially with other anti-PD-1/PD-L1 checkpoint inhibitors. Several antibody clones (especially 22C3, 28-8, SP263, and SP142) were evaluated and showed good reproducibility in harmonization studies [3]. However, in clinical practice, further validation efforts seem necessary since diagnostic reports from various laboratories may be not completely overlapping [4]. The Blueprint project showed that the percentage of PD-L1 positive tumor cells was comparable for clones 22C3, 28-8, and SP263, while clone SP142 characteristically identified lower percentages of positive neoplastic cells [1]. Consequently, the 22C3, SP263, and 28-8 clones are usually chosen by pathologists to test routinely cytological and histological specimens, combining them in close and open commercially available IHC platforms. Moreover, due to the different technical and interpretative expertise, further analytical variables may affect the final local reports [5]. In the Italian scenario, a study confirmed a high correlation between PD-L1 IHC expression data obtained with the 22C3 and SP263 clones, suggesting that the two assays could be utilized interchangeably [2]. After 1 year of PD-L1 routine testing, the present multicentric retrospective study has aimed to compare the results obtained by using different protocols performed on the same tissue microarray (TMA) of a series of NSCLC histological specimens, analyzed in different laboratories and it aimed to evaluate if heterogeneous results still persist, especially when open platforms are used. The data were recorded in terms of interpretative/analytical error, highlighting the current state of reproducibility in the routine practice of PD-L1 IHC test.

## 2. Materials and Methods

Formalin-fixed paraffin-embedded (FFPE) histological samples from 18 lung surgical specimens with a NCSLC were retrospectively selected for this study. The series included adenocarcinomas and squamous cell carcinoma. The inclusion criteria were the following: adult patients (>18 years old) who underwent total or partial pneumonectomy in the period between 1 December 2016 and 31 January 2018 for NSCLC; no previous neoadjuvant chemoradiotherapy was administered. The original samples were recovered from the archive of the Pathology Department of University Milan Bicocca-ASST Monza, San Gerardo Hospital, Monza. The study was approved by the Ethical Committee of ASST Monza, under the approval #N.1311, dated 17/07/2018. To maximize the homogeneity in preanalytical variables, cases were selected from a unique institution with available trackable processing phases. For this study, fixation time was set at 24 hours following the surgical procedure, as previously described [6]. Tissues subsequently were grossed and processed as routine cases; a representative histological hematoxylin and eosin (H&E) stained section of the original nodules was evaluated by two lung-committed pathologists (FB, FP) avoiding little fixed areas of necrosis and fibrosis and the corresponding paraffin block was chosen for the study. For every case a PD-L1 staining (Agilent 22C3 pharmDx on Dako Autostainer, Dako, Glostrup, Denmark) was performed to sample TMA cores, according to three balanced groups: score (1) Tumor Proportion Score (TPS) negative (<1% or absence of reactivity); score (2) intermediate expressors (1-49% of tumor cells); score (3) strong expressors (≥ 50% of tumor cells). For the TMA construction, two separate areas were selected from the original block (about 3 mm in diameter), homogeneous for expression patterns for PD-L1, to be punched using a 2 mm-diameter needle. The TMA layout was built using the Galileo TMA R4.30 ISE software (Integrated Systems Engineering Srl, Milan, Italy). The realization of the TMA blocks was made possible by the use of the semiautomatic ISE Galileo TMA CK 4500 arrayer (Integrated Systems Engineering). Serial sections on positively charged slides of 1-2 micron thickness were obtained. All the collected sections were then kept in a thermostated oven at 60°C overnight. Firstly, TMA blanks were stained using two* closed platforms* to obtain the* gold-standard* scores (Agilent 22C3 PharmDx on Dako Autostainer and Roche's Ventana SP263 on BenchMark with Assay OptiView DAB IHC Detection Kit, Ventana, CA, USA). PD-L1 staining was evaluated by two lung-committed pathologists (FB, FP) in blind and then jointly for the final agreement. Secondly, further TMA blanks were stained using 7 alternative protocols for PD-L1 scoring on* open platforms *(Table 1). The slides were evaluated in blind from the gold-standard results and scoring was recorded in a Microsoft Office Excel 2007 database for the statistical analysis. All the discordant cases were reevaluated jointly by a board team of pathologists to identify the possible source of errors (meeting at UNIMIB in 07/2018).

## 3. Results

**Figure 1:**
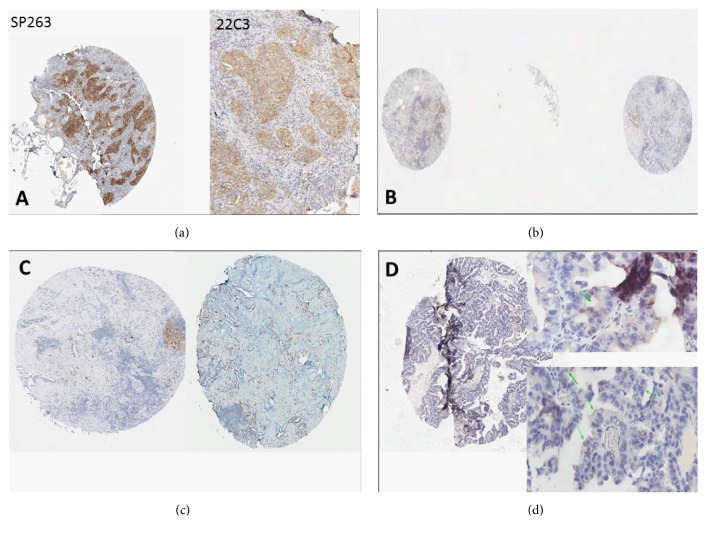
(a) Case 1 (strong expressor); diversity in PD-L1 intensity using the two* gold-standard* methods; by SP263 clone, the positivity was easily evaluated at 4x; by 22C3, pathologists needed to apply a greater magnitude to confirm the reactivity in more than 50% of tumor cells. (b) Technical error. The core was not correctly evaluated due to detachment of the TMA section (case n.10). (c) Technical error. In the picture different levels of the same TMA-sampled core (n.18) hosted heterogeneous PD-L1 expressions. (d) Technical error. Case n.2 evaluated as intermediate expressor using protocol n.6. The board reviewers enhanced a false positive signal in the apex of tumor cells that was not present in the same tissue core whenever stained by other protocols. A similar technical artifact was observed also in case 3 from the same protocol.

In the first phase of the study PD-L1 using two* closed* standard platforms was evaluated (Roche Ventana SP263 on Benchmark and Dako PharmDx 22C3 on Autostainer). In Table 2 the comparative results obtained are listed. In 15 out of 18 cases (83%) PD-L1 scoring overlapped in both cores, using indifferently the two platforms. In 3 out 18 cases (n. 11,12,17) the 22C3/Autostainer PD-L1 staining produced different results in the two cores so the final grade was set on the Ventana staining. The definitive* gold-standard* scores included 6 negative cases and 7 intermediate, and 5 strong expressors. While the conclusive results were equivalent, a certain degree of diversity was noticed in terms of intensity. SP263 produced stronger IHC reactivity, just easily perceptible at 4x; to assess correctly 22C3 staining, a greater magnification than 4x had to be used, in contrast to what happened with SP263 staining, where a low magnification was sufficient (Figure 1(a)). Secondly, the comparison between the* gold-standard* scores and the results obtained by using 7 different* open* platforms were collected in Table 3. All the discordant cases were reevaluated by the expert board team to identify the possible source of error.

Three possibilities were identified.

**Figure 2:**
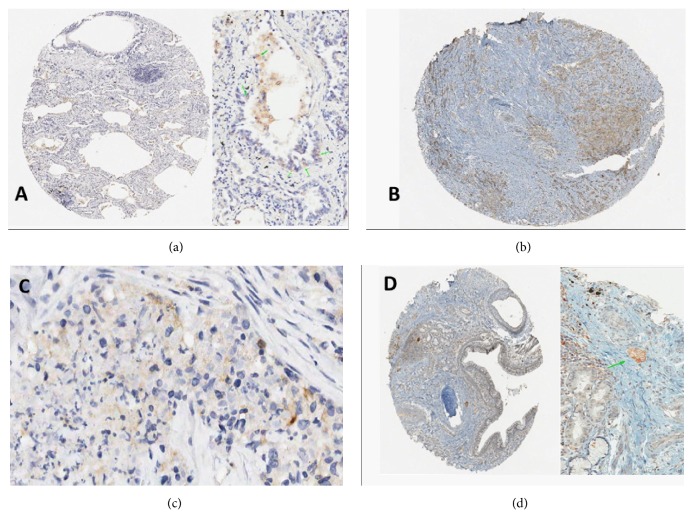
(a) Inappropriate evaluation of immune cells (macrophages) in crushed TMA cores may be a source of error (case 8; gold-standard score 1, wrongly scored as 2). (b) In particular, challenging cases the average threshold of positive tumor cell cells may be wrongly assigned (i.e., 40 versus 60%). (c) In cases (like n.17) with low-intense and focal reactivity only the evaluation at high-power field (40x) may assign correctly as intermediate expressor the tumor, differentiating immune and neoplastic cells. (d) If a TMA core is sampled at the periphery of the main tumor nodule, where only occasional embolic aggregates are present, they may be disregarded by a not accurate pathological examination (case n.12).

1. Technical errors (T): exemplificative situations are listed in Figures 1(b)–1(d).
2. Pathologist interpretative errors (P): typical examples are shown in Figures 2(a)–2(d).
3. Mixed errors (M): in these cases, a combination of low intensity due to technical reasons (compared to* gold-standar*d) and underestimated signal by the pathologists produced the error.

A total of 23 out of 126 tests (18%; 4T, 15P, 4 M) were affected by the three error sources, globally; error rate (ER) of the single centers ranged from 25% (5/ 18) to 5% (1/18). Sensitivity ranged from 69% to 92% and specificity from 33% to 100%; the best performance was obtained by protocol n.2 using clone 22C3 on Dako Omnis (ER=5.5%; Sn=92%; Sp=100%) and protocols n.3,7 (ER=11%; Sn=85%; Sp=100%) using clone 22C3 on Dako Omnis and Ventana Benchmark, respectively (Table 4).

### 3.1. Negative Cases and Therapy (N=6)

Five out of 7 protocols assigned correctly all the negative cases (Table 5). For 2 protocols the board pointed out in the study possible problems in terms of specificity, due to prevalent interpretative errors. In 2 out of 6 patients PD-L1 testing was negative independently from the platform and the center performing the examination.

### 3.2. Intermediate Expressor Cases and Therapy (N=7)

In the intermediate group a certain disagreement persisted; however technical (or mixed) errors seemed to be more relevant than in negative cases. Two out 10 errors were scored as strong expressors instead of intermediate; in the majority of the situations the score was underestimated (Table 6).

### 3.3. Strong Expressor Cases and Therapy (N=5)

The positive group had a good diagnostic agreement for technical and interpretative variables (Table 7). Case n.12 (Figure 2(d)) was particularly challenging (isolated tumor cells in normal lung parenchyma), highlighting the importance of a high magnification examination in absence of strong PD-L1 signal.

## 4. Discussion

Immune-checkpoint inhibitors have changed the treatment paradigm in locally/advanced NSCLC [7–9]. There are four monocolonal antibodies that are currently used in clinical practice, with some overlapping indications: nivolumab in pretreated patients [10] as well as atezolizumab [11], pembrolizumab that extends the possibility of using upfront immune-checkpoint inhibitors (ICIs) [12], and more recently durvalumab as maintenance/consolidative treatment in patients with nonoperable locally advanced NSCLC who benefit from chemoradiotherapy [13]. All these drugs have an indication more or less linked to tumoral IHC PD-L1 expression. In particular pembrolizumab was granted in first line setting only in tumors that express a strong PD-L1 tumor proportion score (TPS≥50%) while in further lines of therapy a positivity of PD-L1 (≥1%) is sufficient to indicate its employment [14]. The same situation regarding durvalumab, after a recent post hoc analysis in which the maximum benefit is demonstrated in PD-L1 ≥ 1%, keeps the indication after chemoradiation in these tumors alone [13]. Nivolumab and atezolizumab have the indication from second line of treatment in all comers without restriction of PD-L1 tumor expression. In this scenario how the detection and correct interpretation of PD-L1 expression on tumor cells are crucial in order to allow the patients the best therapeutic strategy becomes evident [15]. Beyond the technical aspects discussed below it is important to note the intra- and intertumoral heterogeneity of PD-L1 expression that may affect the reproducibility of this analysis [16]. Finally, the conservation of archived tissue samples may impact the detection and staining degree leading to misinterpretation of PD- L1 status [17–19]. This multicentric retrospective study evaluated the reproducibility of PD-L1 testing in the Italian scenario both for* closed* and* open* platforms. In the evaluation of the well-known* gold-standard* combinations (Agilent 22C3 PharmDx on Dako Autostainer versus Roche's Ventana SP263 on BenchMark), the results confirmed the literature data and showed complete overlapping between the two methods. As regards the intensity levels of the staining, the use of the Ventana platform produced more intense reactions in the face of a morphological more difficult distinction between the tumor cells and the immune cells (usually alveolar macrophages, normally positive) while the Dako platform provided fairly soft reactions but with morphological differentiation of the most obvious cell types. Secondly, the comparison between the* gold-standard* and the PD-L1 IHC staining obtained by using 7 alternative locally validated protocols on open platforms reflected some possible sources of errors in the routine practice. The study identified mainly two exemplificative situations: interpretative errors (Figure 2) that affected basically false negative results and technical ones (Figure 1). In the first group the possible staining of the immune cells in the histological sample may complicate the histological interpretation as the inappropriate application of the magnification rule that recommends the use of high-power field for the scoring of mild/ focal reactivity. As shown in Table 6, in the intermediate group the sensitivity may be affected whenever a mild or focal staining, related to a low technical amplification or a low antigen retrieval, is a source of false negative interpretative errors. On the other hand, a low specificity is also possible, as in protocols 4 and 6 (Table 5) due to the difficulties of pathologists in distinguishing macrophages from neoplastic cells in particularly challenging specimens or avoiding false positive staining in mucus-rich tumors. In this subgroup, the study revealed as some protocols may frequently produce unspecific perimembranous reactivity. Every laboratory should set the proper PD-L1 protocol to avoid this signal that is inappropriately considered as positive.

Another difficult situation was pinpointed when pathologists should decide around the threshold of 50% positive cells, suggesting that only a careful and extensive quantitative evaluation may avoid an underestimation in the real routine practice. Among the strong positive patients only one (case 12) produced equivocal results, due to a particularly challenging TMA core that included isolated (PD-L1 positive) tumor cells. For the technical errors, a significant proportion of them may be related to the limitations of a TMA-based study; the heterogeneity in the serial levels of the histological sections may in fact exclude focal PD-L1 positive foci from the analysis. Moreover, due to TMA intrinsic characteristics, some cores cannot be adequately examined (Figure 1). With regard to the performances by using* open* platforms, the combination of 22C3 with Dako Omnis or Benchmark obtained good results basically, while the 28,8 clone seemed to be associated with worse scores.

## 5. Conclusions

This study was designed to stress the methodological challenges of the PD-L1 IHC testing and collected particularly difficult cases by a preliminary histological selection of NSCLC samples that did not reflect necessary a normal case-mix. By these limitations, we can conclude that oncologists should remember that the bioselection of NSCLC patients by the PD-L1 staining has still some technical and interpretative* caveat.* On the other hand, after several efforts in order to harmonize the read-out lecture of PD-L1 status among different antibody clones, assays, and platforms, pathologists have now focused experiences and adequate training to give more detailed and reproducible PD-L1 results to clinicians [20, 21].

## Figures and Tables

**Table 1:** Technical protocols in gold standard and open platforms of the study.

PROTOCOLS	CLONE	PLATFORM	TITER	PH	Incubation primary Ab	Incubation linker/polymer
Gold standard 1	22C3	Autostainer	PharmDx prediluted	low 20′-97°	30′	30′/30′

Gold standard 2	SP263	Ventana	prediluted	(CC1) High pH ETDA retrieval buffer	16′- 37°	-

1	28-8	Dako Omnis	1:500	high 30′-97°C	20′	10′/20′

2	22C3	Dako Omnis	1:20	low 40′ - 97°	40′	10′/40′

3	22C3	Dako Omnis	1:20	low 40′ - 97°	40′	10′/40′

4	22C3	Dako Omnis	1:50	low 30′ - 97°	30′	10′/30′

5	22C3	Dako Omnis	1:50	low 30′ - 97°	30′	10′/30′

6	22C3	Dako Omnis	1:20	low 40′ - 97°	40′	20′/40′

7	22C3	Ventana	1:40	(CC1) High pH ETDA retrieval buffer	32′ - 36°	12′ with OptiView DAB IHC detection kit

**Table 2:** PD-L1 results of the TMA-based analysis using closed platforms.

	Case 1	Case 2	Case 3	Case 4	Case 5	Case 6

22C3/Autostainer	3	1	1	2	3	3

SP263/BenchMark	3	1	1	2	3	3

*FINAL SCORE*	*3*	*1*	*1*	*2*	*3*	*3*

	Case 12	Case 11	Case 10	Case 9	Case 8	Case 7

22C3/Autostainer	2- 3	1-2	2	1	1	2

SP263/BenchMark	3	2	2	1	1	2

*FINAL SCORE*	*3*	*2*	*2*	*1*	*1*	*2*

	Case 13	Case 14	Case 15	Case 16	Case 17	Case 18

22C3/Autostainer	2	1	3	1	1-2	2

SP263/BenchMark	2	1	3	1	2	2

*FINAL SCORE*	*2*	*1*	*3*	*1*	*2*	*2*

**Table 3:** Comparison between the gold standard and the PD-L1 results on open platforms.

	1	2	3	4	5	6	7	8	9	10	11	12	13	14	15	16	17	18	Sn	Sp
1				M			M			T		P						T	69%	100%

2												P							92%	100%

3												P						M	85%	100%

4			P					P				P		P					92%	50%

5												P	M				P	P	69%	100%

6		T	T					P				P		P					92%	33%

7											P						P		85%	100%

GOLD	3	1	1	2	3	3	2	1	1	2	2	3	2	1	3	1	2	2		

**Table 4:** Correlation between open platforms and type of errors.

PROTOCOLS	CLONE	PLATFORMS	ERRORS
1	28-8	Dako Omnis	4(2M; 1T; 1P)

2	22C3	Dako Omnis	1(P)

3	22C3	Dako Omnis	2 (1P,1M)

4	22C3	Dako Omnis	4(4P)

5	22C3	Dako Omnis	4(3P,1M)

6	22C3	Dako Omnis	5(3P,2T)

7	22C3	Ventana	2(2P)

**Table 5:** Errors in PD-L1 negative cases.

	cases					

protocols	2	3	8	9	14	16

1						

2						

3						

4		P	P		P	

5						

6	T	T	P		P	

7						

GOLD	1	1	1	1	1	1

**Table 6:** Errors in PD-L1 intermediate expressors.

	cases						

protocols	4	7	10	11	13	17	18

1	M	M	T				T

2							

3							M

4							

5					M	P	P

6							

7				P		P	

GOLD	2	2	2	2	2	2	2

**Table 7:** Errors in PD-L1 strong expressors.

protocol	1	5	6	12	15
1				P	

2				P	

3				P	

4				P	

5				P	

6				P	

7					

GOLD	3	3	3	3	3

## Data Availability

The immunohistochemical data used to support the findings of this study are included within the article.
